# Does combined cognitive training and physical activity training enhance cognitive abilities more than either alone? A four-condition randomized controlled trial among healthy older adults

**DOI:** 10.3389/fnagi.2013.00008

**Published:** 2013-03-26

**Authors:** Evelyn Shatil

**Affiliations:** ^1^CogniFit Inc.New York, NY, USA; ^2^The Center for Psychobiological Research, Max Stern Academic College of Emek YezreelJezreel Valley, Israel

**Keywords:** older adults, cognitive training, cognitive function, physical activity, CogniFit, brain plasticity

## Abstract

Cognitive training and aerobic training are known to improve cognitive functions. To examine the separate and combined effects of such training on cognitive performance, four groups of healthy older adults embarked on a 4 months cognitive and/or mild aerobic training. A first group [*n* = 33, mean age = 80 (66–90)] engaged in cognitive training, a second [*n* = 29, mean age = 81 (65–89)] in mild aerobic training, a third [*n* = 29, mean age = 79 (70–93)] in the combination of both, and a fourth [*n* = 31, mean age = 79 (71–92)] control group engaged in book-reading activity. The outcome was a well-validated multi-domain computerized cognitive evaluation for older adults. The results indicate that, when compared to older adults who did not engage in cognitive training (the mild aerobic and control groups) older adults who engaged in cognitive training (separate or combined training groups) showed significant improvement in cognitive performance on Hand-Eye Coordination, Global Visual Memory (GVM; working memory and long-term memory), Speed of Information Processing, Visual Scanning, and Naming. Indeed, individuals who did not engage in cognitive training showed no such improvements. Those results suggest that cognitive training is effective in improving cognitive performance and that it (and not mild aerobic training) is driving the improvement in the combined condition. Results are discussed in terms of the special circumstances of aerobic and cognitive training for older adults who are above 80 years of age.

## Introduction

With increasing age, older adults' cognitive ability declines (Salthouse, 2004). Research shows declining ability in working memory, long-term memory (Park et al., 2002); dual-tasking, task-switching (Verhaeghen and Cerella, 2002), reasoning ability (Schaie, 1996), processing speed, and executive and attentional control (Salthouse, 2004). As a result, individuals' quality of life, autonomy, and daily activities such as driving, scheduling the taking of medications, and grocery shopping may be negatively impacted (Owsley et al., 1998; Wadley et al., 2008). Many possible strategies to preserve or enhance cognitive function in older adults have been investigated. Evidence based on numerous investigations suggests that two kinds of interventions, cognitive training and physical activity training, enhance cognitive function in healthy older adults (review by Jak, 2011). Scant research, however, exists on their combined effectiveness.

Research on older adults has linked physical activity to several physiological (Anderson et al., 2010) and neurological (Motl et al., 2008) gains and to increases in longevity (Erikssen et al., 1998; Hu et al., 2004). Physical activity has also been attributed important protective (Colcombe et al., 2003) and regenerative (Colcombe et al., 2006) functions against cognitive decline. Aerobic physical activity interventions (mostly swimming or brisk walking) have been associated with improved attention (Colcombe et al., 2004) and executive control processes such as switching and inhibition (Kramer et al., 1999; Colcombe and Kramer, 2003). Such improvements are considered specific to the frontal and parietal cortex as increased activation in those areas was observed in older adults who were either highly fit or after a 6-month training regimen (Colcombe et al., 2004). Aerobic training was shown also to reverse brain volume loss (Colcombe et al., 2006). Conversely, a decrease in executive function in older adults is associated with impaired gait and mobility (Holtzer et al., 2006, 2007).

Similarly, cognitive training interventions such as computer-based exercises that offer practice in memory, attention, fine-motor coordination, visual and auditory processing have been associated with cognitive gains in healthy older adults (Ball et al., 2002; Willis et al., 2006; Bherer et al., 2008), which may last for several years (Ball et al., 2002; Willis et al., 2006). The majority of studies suggest that cognitive improvements do not transfer to new tasks or transfer only to tasks with the same processing requirements as the trained tasks (Ball et al., 2002; Mahncke et al., 2006; Basak et al., 2008; Bherer et al., 2008; Li et al., 2008; Smith et al., 2009; Owen et al., 2010; Verghese et al., 2010), with the effect sizes for a cognitive intervention being quite small (review by Papp et al., 2009).

Cognitive training may target multiple cognitive domains (Shatil et al., 2010; Verghese et al., 2010) or a single one such as memory (Mahncke et al., 2006), attentional control (Bherer et al., 2008), linguistic verbal-auditory processing (Smith et al., 2009), or working memory (Horowitz-Kraus and Breznitz, 2009). Interventions may vary on minimum number, frequency, and length of training sessions. They may be administered in person by a technician using oral instruction and practice (Ball et al., 2002) or via computer (Smith et al., 2009; Verghese et al., 2010; Peretz et al., 2011). With regard to computerized interventions, it remains unclear which types might improve cognitive ability the most (Thompson and Foth, 2005). It appears reasonable that training approaches designed to accommodate each individual's current neuropsychological strengths and weaknesses, as well as those providing instant item-specific feedback (Bherer et al., 2008; Kramer and Morrow, in press) and dynamically adapting the training program accordingly, would be especially effective, particularly among people with particular cognitive enhancement needs (Faucounau et al., 2010).

Only two publications have reported on studies comparing separate effects of cognitive and physical activity training to those of the combined training (Fabre et al., 2002; Oswald et al., 2006). The former compared aerobic training, mental training, and combined aerobic-mental training among 32 healthy older adults aged 60–76 years. Significant post-training improvements were observed in story memory, paired-associate learning and memory quotient in the three trained groups. The mean difference in memory quotient between pre- and post-training was significantly higher in the combined training group compared to either of the other two groups. The latter evaluated the longitudinal effects of mental, physical, and combined training 1 year and 5 years after training, in a sample of 375 healthy, independently living older adults aged 75–93 years. The mental intervention had a significant paper and pencil training component and targeted speed of information, memory storage and retrieval, attention and compensatory strategies using everyday life materials. The physical intervention targeted balance, flexibility, and motor coordination using group exercises and games, some of them requiring accelerated responses. Effect sizes in the cognitive training condition and in the combined condition were superior across a range of cognitive outcomes 1 year after training and still evident 5 years later. Physical activity alone was associated with no cognitive advantage at either follow-up.

The present study sought to replicate and extend, in a population of healthy older adults, findings from the two earlier studies using combined interventions, as well as from other cited studies evaluating cognitive or physical training separately. Using a multi-domain neuropsychological evaluation as an outcome measure, when compared to an active control group, we expected a physical activity group to show improvements in attentional and executive function processes (Kramer et al., 1999; Colcombe et al., 2004); a cognitive training group to show improvements on speed of information processing (Ball et al., 2002), visuospatial working memory, learning, and focused attention (Peretz et al., 2011); and a combined intervention group to display improvements on the same cognitive abilities as both the single intervention groups. Moreover, we expected cognitive improvements to be superior for the combined intervention, due to its twice-longer duration and to its possibly additive or multiplying benefits.

Whereas both combined intervention studies discussed employed programs and activities requiring a significant amount of human resources (teachers, coaches) to implement, the present study employed computerized, individually adapted technology. Thus, an additional goal of this study was to document the intervention's implementation in real-world community settings, especially as regards adherence. To achieve this goal we created a unique partnership between an academic research setting, a retirement community, and two industry members—a cognitive fitness enterprise and a physical fitness enterprise—who provided the scientific know-how, the study site and manpower, and the intervention programs, respectively.

## Methods

Using a randomized controlled four-group design (cognitive intervention, physical activity intervention, combined cognitive-physical activity intervention, and book reading and discussion control group), we attempted to evaluate the efficacy of cognitive training, physical activity training, and both combined to improve cognitive function in healthy seniors.

### Research collaboration and roles

Four partners collaborated on this study. The Lakeview retirement community of Lenexa, KS, United States (http://www.lakeviewvillage.org/) provided the site for the study, donated two principal coordinators for the main interventions (physical activity and cognitive training) medical staff and volunteers from the community, all of whom were involved in the logistics of the project's implementation and erected an applied research center with modern facilities for cognitive and physical activity training using in kind-donations and tax exemption for the expenses incurred. The Fitness Forever™ Senior Exercise Video served as the primary component of the physical activity intervention and was donated by Life Span Fitness Ltd. (http://www.fitnessforever.com/). CogniFit Ltd. (http://www.cognifit.com/) donated the cognitive training program, *CogniFit*®, and provided the scientific and technologic platform for collecting, processing, and analyzing the neurocognitive performance data. Finally, the Beckman Institute for Advanced Science and Technology at the University of Illinois contributed to the planning of the study and provided training to the Lakeview staff on applying the intervention and collecting the data.

### Participants and procedure

Participants were healthy volunteers residing at the Lakeview retirement community of Lenexa, KS. They were recruited via paper fliers, e-mails, telephone calls, and during an “information day” attended by the four study partners, and including presentations on the circumstances of the study as well as demonstrations of the various technologies. Exclusion criteria included having suffered a stroke or heart attack in the past 5 years, taking drugs that might adversely affect cognition (including benzodiazepines and antipsychotics), or scoring ≤23 on the Mini Mental State Examination (MMSE). Inclusion criteria included having a corrected vision of at least 20/40, ability to clearly hear instructions, and communicate with experimenters, agreeing to participate in the assessment and training sessions throughout the course of the study, completing a medical history, and obtaining a physician's recommendation.

Residents who were willing to participate in the study gave informed consent on the information day and were subsequently interviewed to verify that they were not already engaged in physical or cognitive training. Eligible participants were then screened on the MMSE to ascertain a score of ≥24. Following the screening subjects were randomized to the four intervention groups.

The study protocol was approved by a private institutional review board (Independent Review Consulting, Inc., San Anselmo, CA), specializing in non-university trials. Study participants did not receive any monetary compensation but were provided with complimentary cognitive training programs at the end of the study. Prior to the onset of the study both research coordinators and adjunct staff took the course Collaborative Institutional Training Initiative (CITI). (https://www.citiprogram.org/).

### The interventions

The four interventions were conducted in groups of approximately 15–20 participants at the Applied Research Center at Lakeview. All meetings afforded a significant amount of social interaction. Residents chose their training days and times based on pre-scheduled group sessions for their group. However, when the need arose participants were allowed to replace their scheduled session with another session in the same week. Adherence to the training activities was closely monitored by the study coordinators. Support and encouragement for perseverance were provided by a group of Lakeview residents who had volunteered for this function.

#### The cognitive training intervention

CogniFit, the program used in this study, has been described and validated in prior studies (Horowitz-Kraus and Breznitz, 2009; Shatil et al., 2010; Verghese et al., 2010; Peretz et al., 2011; Haimov and Shatil, in press). Participants in the single and combined interventions trained for a total of 32 h arranged in 48 forty-minute sessions (three times weekly for 16 weeks) with at least a 1-day interval between sessions.

In each session, the first 20 min were spent training on combinations of three of the 21 tasks in the CogniFit personalized regimen described in previous work (Shatil et al., 2010; Verghese et al., 2010; Peretz et al., 2011). For the remaining 20 min participants practiced any three tasks of their own choice among the 21 tasks.

#### The physical activity intervention

59% of all participants were 80 years or older (range 65–93) and could not deal with intensive aerobic training. Therefore The Fitness Forever™ Senior Exercise Video was selected for the study. Each session included aerobic warm-up (10 min), cardiovascular workout seated and standing (15 min), aerobic cool-down (5 min), strength training (10 min), and flexibility training (5 min). Training was followed by brief relaxation. Individuals that were not fit enough to manage initially a 45 min exercise duration began with a medically appropriate exercise program and built up to the 45 min requirement. Participants exercised in a classroom, group format. Participants were checked-in into the class and provided chairs to relax in before the class began, and to use during the class as needed. They were taught and required to monitor their Rate of Perceived Exertion (RPE) and their heart rate at the completion of each exercise session. The exercise instructor-assisted participants with exercise form, posture, and anything else needed during the exercise class. A portable automated external defibrillator (AED) was also available in the exercise room, along with a phone for calling an emergency code should an emergency situation arise. RPE charts were placed in the exercise room for reference during the exercise sessions. The training, which was displayed on a large television screen and led by a qualified fitness instructor also trained in emergency procedures, consisted of three weekly 45 min sessions, with at least a 1-day interval between training days, during 16 weeks.

#### The combined cognitive training and physical activity intervention group

Participants in this group were required to undergo both the cognitive training and the physical activity training interventions described in the above sections. These participants received twice as many training sessions as did the cognitive or physical activity training participants.

#### The Book Club Control group

Participants in this group read the book “Active Living Everyday: Twenty Weeks to Lifelong Vitality” (Blair et al., 2001). For the duration of the study, this group was assigned selected book excerpts to be read at home and held one 60 minutes weekly meeting during which the best ways to achieve the goals advocated in the book were discussed.

### Outcome: cognitive function

To measure change in cognitive function following the interventions, we used the CogniFit neuropsychological evaluation, requiring three 15-min sessions to administer. It is composed of 15 evaluation tasks measuring a wide range of cognitive abilities such as focused and divided attention, inhibition, shifting, planning, working memory, and eye-hand coordination. Scores are derived from response times (in milliseconds) and accuracy (%). Raw data collected from the tasks are reduced and scores assigned to 17 traditionally recognized cognitive abilities using weights previously derived from a factor analysis performed on data from a healthy population (*N* = 861, 517 females and 344 males, average age 65.7 ± 8.85 years, range 50–90 years).

The CogniFit neuropsychological evaluation has been validated in healthy younger adults (mean age 23 years) against major standard neuropsychological tests, including the full Cambridge Neuropsychological Test Automated Battery, Raven's Standard Progressive Matrices, the Wisconsin Card Sorting Test, the Continuous Performance Test, the STROOP test, and other tests (Haimov et al., 2008). Tests of its reliability were also undertaken using data from a study of 89 participants aged 50 and over, yielding adequate measures of internal consistency (Chronbach's alpha = 0.70) and test—retest reliability (intra-class correlation coefficient = 0.80) (Haimov et al., 2008). Its scores correlate with well-being and spirituality (Thompson et al., 2011) and it has been used as an outcome measure in previous research (Shatil et al., 2010).

## Results

### Adherence

#### Adherence after informed consent

On the information day, 216 subjects signed an informed consent form to participate in the study, but shortly thereafter, and before subjects' screening for inclusion began, 36 (16.7%) re-considered and cancelled their intention to participate. Reasons for retracting are listed in Table 1 and included surgery, pain, vision and hearing problems, hospitalization; engagement with own hobbies, activities, and family obligations; care-giving to sick husband or wife and computer stress.

**Table 1:** **Reasons evoked for retraction after informed consent**.

**Reasons**	***N*% out of 36 subjects**	***N*% out of 216 subjects**
Heavy personal activity load	10 (27.8)	10 (4.6)
Poor health	16 (44.4)	16 (7.4)
Caregiver for sick husband or wife	4 (11.1)	4 (1.9)
Computer stress	2 (5.6)	2 (0.9)
Unknown	4 (11.1)	4 (1.9)
Total	36 (100)	36 (16.7)

#### Adherence during baseline testing

As the 180 recruited elder subjects fell in the MMSE inclusion range and were able to obtain medical recommendation, they were randomly assigned to the four study groups. Among those, 55 participants (30.5%) left during the baseline testing period, while another battery of tests (to be reported elsewhere) were being administered; before the training interventions had begun and before the particular computerized battery used as a cognitive outcome for this study had been administered.

#### Adherence during the intervention

Three participants, two in the Cognitive Training Group and one in the Physical Activity Group, left the study, due to health problems. Thus, altogether, 58 subjects (32.2% among the 180 enlisted study participants) withdrew from the study and 122 adhered to it (31 in the Physical Intervention Group, 33 in the Cognitive Intervention Group, 29 in the Combined Intervention Group, and 29 in the Book-Reading Intervention Group).

Table 2 shows adherence patterns for the four groups. Although adherence differences between the groups did not reach statistical significance (χ^2^ = 1.883, *p* = 0.597), the number of non-completers was greatest in the combined intervention group (39.6%) and smallest in the cognitive training group (26.7%).

**Table 2:** **Adherence rates in the four study groups**.

**Recruited subjects**			
**Group**	**Completers *N* (%)**	**Non-completers *N* (%)**	**Total *N***
Physical activity	31 (68.9)	14 (31.1)	45
Cognitive training	33 (73.3)	12 (26.7)	45
Combined training	29 (60.4)	19 (39.6)	48
Book reading	29 (69.0)	13 (31.0)	42
Total	122 (67.8)	58 (32.2)	180

Table 3 describes the reasons for non-adherence by intervention group for the 58 non-completers.

**Table 3:** **Reasons for non-adherence by intervention group**.

	**Physical activity**	**Cognitive training**	**Combined training**	**Book-club control**	**Total**
	***N* (%)**				
Personal load	6 (42.9)	5 (41.7)	6 (31.6)	2 (15.4)	19 (32.8)
Poor health	6 (42.9)	2 (16.7)	7 (36.8)	7 (53.8)	22 (37.9)
Main caregiver		2 (16.7)	1 (5.3)	3 (23.1)	6 (10.3)
Dissatisfaction with study	1 (7.1)		3 (15.8)		4 (6.9)
Unknown	1 (7.1)	3 (25)	2 (10.5)	1 (7.7)	7 (12.1)
Total	14 (100)	12 (100)	19 (100)	13 (100)	58 (100)

Differences in patterns of non-adherence between the groups did not reach statistical differences (χ^2^ = 12.449, *p* = 0.189). Health-related problems, such as planned surgery, recovery from surgery, sight and hearing problems, and hospitalization, were the most prominent reason for leaving the study (22 participants, 37.9% of non-completers); while heavy personal activity load such as dancing, traveling, and gardening which proved incompatible with the time requirements of the study (19 participants, 32.8% of non-completers) was also a major reason. Less common reasons for leaving the study were unexpectedly becoming the sick husband's or wife's main caregiver during the study (6 participants, 10.3% of non-completers) and dissatisfaction with aspects of the study such as not being assigned to a preferred intervention group or being frustrated with the large amount of testing at the onset of the study, (4 participants, 6.9% non-completers). Note that whereas 36.8, 42.9, and 53.8% left the combined, physical, and book-reading interventions respectively, relatively fewer participants (16.7%) invoked health issues for leaving the cognitive training intervention.

We compared the 58 subjects who did not complete the study to the 122 who did, on gender, age, and years of education. Results of analyses are presented on the education variable, although data was unavailable for 48.2% of the non-completers, on this variable. Table 4 shows significant differences for age and years of education but not for gender distribution, between participants who did not complete the study and those who did.

**Table 4:** **Demographic attributes of completers and non-completers by intervention group**.

	**Completers (*n* = 122)**		**Non-completers (*n* = 58)**		***T*-statistics**		
	***M***	***SD***	***M***	***SD***	***M*-difference**	***t***	***p***
**CHARACTERISTICS**							
Age	76.83	5.51	81.02	4.93	−4.2	−4.93	0.000
Years of education (*N* = 30 for non-completers)	15.70	2.43	14.48	1.88	1.2	2.55	0.012
	***N***	**%**	***N***	**%**		**χ^2^**	***p***
**GENDER**							
Female	84	68.94	44	76		0.382	0.215

Non-completers were significantly older, on average by 4 years, than completers, and had on average 1.2 years less formal education. Further analyses performed for each intervention group separately indicated that non-completers were significantly older in the physical activity group (age difference = 5.3 years; *t* = −3.024, *p* = 0.004), in the cognitive training group (4.1 years, *t* = −2.306, *p* = 0.026) and in the combined intervention group (4.3 years, *t* = −2.651, *p* = 0.011) but not in the control group (3.5 years, *t* = −1.791, *p* = 0.081). They were significantly less educated in the physical activity group (difference in years of education = 2 years, *t* = −2.124, *p* = 0.04) only.

### Participants

Table 5 shows that, at the onset of the study, except for education, the four study groups (Total *N* = 125) were similar on a large number of personal attributes. Further comparisons clarified that the combined intervention group was less educated than the other groups, although this difference reached statistical significance only in the comparison with the physical activity intervention group. Education was, therefore treated as a covariate in the between-group comparisons reported later on.

**Table 5:** **Comparisons by group of the personal characteristics of participants at baseline**.

	**Cognitive training**				**No cognitive training**					
	**No physical activity (*n* = 33)**		**Physical activity (*n* = 29)**		**No physical activity (*n* = 29)**		**Physical activity (*n* = 31)**			
	***M***	***SD***	***M***	***SD***	***M***	***SD***	***M***	***SD***	***F*_(3, 120)_**	***p***
**CHARACTERISTICS**										
Age in years	80	5.43	79	5.49	81	5.25	79	5.76	0.827	0.482
	***N***	**%**	***N***	**%**	***N***	**%**	***N***	**%**	**χ^2^**	***p***
**GENDER**										
Female	23	69.7	20	69	19	65.5	22	71	0.226	0.973
**EDUCATION**										
Some college and above	26	78.8	17	58.6	23	79.3	28	90.3	8.798	0.032^*^
**HEALTH**										
Very good	19	57.6	18	62.1	10	34.5	18	58.1	11.321	0.079
Good	11	33.3	11	37.9	15	51.7	13	41.9		
Poor	3	9.1	0	0	4	13.8	0	0		
**COMPUTER USE**										
Very often	14	42.4	13	44.8	9	31	13	41.9	6.697	0.669
Often	11	31.4	7	24.1	8	27.6	9	29.0		
Seldom	2	6.1	5	17.2	4	13.8	6	19.4		
Never	6	18.2	4	13.8	8	27.6	3	9.7		
**COLOR BLINDNESS**										
None	32	97.0	28	96.6	25	86.2	30	96.8	4.569	0.206
**DOMINANT HAND**										
Right hand	30	90.9	27	93.1	27	93.1	31	100	2.717	0.437

* *p significant at the 0.05 level*.

### Cognitive scores

Correlations were calculated among the 17 cognitive ability scores yielded at baseline by the CogniFit neuropsychological assessment. Table 6 shows high inter-correlations (*p* > 0.80) among three memory variables (Visual Memory, General Memory, and Working Memory), as well as among three other variables (Reaction Time, Spatial Perception, and Visual Perception). Therefore, for each cluster one single score was formed by averaging the three ability scores in the cluster. Inspection of the abilities in the clusters suggests that the first cluster represents global visual memory (GVM) which encompasses visual working-memory and long-term memory while the second cluster represents visual and spatial processing with the emphasis on the speed at which these processes are carried out. These “integrated” abilities are called in this study GVM and Speed of Visual-Spatial Information Processing (SVP).

**Table 6:** **Correlations among the 17 cognitive abilities in the CogniFit evaluation at baseline**.

	**AM**	**AW**	**DA**	**DS**	**GC**	**GM6**	**IN**	**NM**	**PL**	**RT**	**SH**	**SP**	**TE**	**VM**	**VP**	**VS**	**WM**
AM	1	−0.171	0.212^*^	0.174	0.526^**^	0.390^**^	−0.310^**^	0.565^**^	−0.154	−0.683^**^	0.405^**^	0.593^**^	0.771^**^	0.395^**^	0.695^**^	−0.507^**^	0.301^**^
AW		1	0.067	0.087	0.000	−0.083	0.018	0.017	0.046	0.076	−0.041	−0.099	−0.040	−0.052	−0.163	0.031	−0.097
DA			1	0.688^**^	0.341^**^	0.261^**^	0.042	0.465^**^	−0.045	−0.376^**^	0.205^*^	0.328^**^	0.179^*^	0.298^**^	0.314^**^	−0.324^**^	0.268^**^
DS				1	0.356^**^	0.256^**^	−0.274^**^	0.612^**^	−0.110	−0.308^**^	0.300^**^	0.321^**^	0.159	0.284^**^	0.239^**^	−0.321^**^	0.231^**^
GC					1	0.645^**^	−0.231^**^	0.649^**^	−0.148	−0.758^**^	0.523^**^	0.760^**^	0.582^**^	0.655^**^	0.680^**^	−0.627^**^	0.602^**^
GM						1	−0.309^**^	0.547^**^	−0.385^**^	−0.498^**^	0.453^**^	0.619^**^	0.433^**^	0.984^**^	0.548^**^	−0.466^**^	0.978^**^
IN							1	−0.405^**^	0.232^**^	0.142	−0.322^**^	−0.157	−0.257^**^	−0.286^**^	−0.160	0.097	−0.315^**^
NM								1	−0.227^*^	−0.695^**^	0.553^**^	0.663^**^	0.550^**^	0.568^**^	0.707^**^	−0.538^**^	0.507^**^
PL									1	0.086	−0.271^**^	−0.198^*^	−0.189^*^	−0.373^**^	−0.186^*^	−0.069	−0.371^**^
RT										1	−0.447^**^	−0.910^**^	−0.706^**^	−0.550^**^	−0.891^**^	0.741^**^	−0.427^**^
SH											1	0.426^**^	0.432^**^	0.463^**^	0.521^**^	−0.357^**^	0.412^**^
SP												1	0.621^**^	0.650^**^	0.800^**^	−0.724^**^	0.572^**^
TE													1	0.457^**^	0.715^**^	−0.513^**^	0.344^**^
VM														1	0.592^**^	−0.505^**^	0.953^**^
VP															1	−0.662^**^	0.477^**^
VS																1	−0.377^**^
WM																	1

*AM, auditory (non-linguistic) working memory; AW, self-awareness; DA, divided attention; DS, avoiding distracters; GC, hand-eye coordination; GM, general memory; IN, inhibition; NM, naming; PL, planning; RT, response Time; SH, shifting; SP, spatial perception; TE, time estimation; VM, visual working memory; VP, visual perception; VS, visual scanning; WM, verbal auditory working memory*.

* p = 0.05;

** *p = 0.01*.

### Data analysis for group differences

We used the General Linear Models for Repeated Measures in the SPSS statistical software (version 18, 2009) to investigate effects of the interventions, on each of the final 13 cognitive abilities. The within-subject variable was TIME and had two levels (baseline and post-intervention ability score). There were two between-subject variables, COGNITIVE and PHYSICAL and each had two levels, trained or did not train. This model allowed for the examination of the two-way (TIME × COGNITIVE and TIME × PHYSICAL) and three-way (TIME × COGNITIVE × PHYSICAL) interactions that would specify which intervention led to cognitive improvements and for whom.

#### Between group differences

Table 7 indicates that the four groups of participants who completed the study (*N* = 122) were similar at baseline on all cognitive ability scores, Table 8 shows the baseline, post-training means and standard deviations of those participants and Table 9 presents the statistics for the interaction effects in the between-group comparisons, with education held as a covariate.

**Table 7:** **Baseline means, standard deviations, and one-way analyses of variance for the four groups on 13 cognitive abilities**.

		***M***	***SD***	***F***	***p***
AM	Physical activity (*n* = 31)	−0.28	0.93	0.351	0.789
	Cognitive training (*n* = 33)	−0.06	0.93		
	Combined training (*n* = 29)	−0.21	0.82		
	Book club (*n* = 29)	−0.26	1.17		
AW	Physical activity	0.04	1.02	1.751	0.160
	Cognitive training	−0.39	0.92		
	Combined training	−0.07	1.03		
	Book club	−0.42	0.91		
DA	Physical activity	1.76	1.03	0.593	0.621
	Cognitive training	1.57	1.26		
	Combined training	1.92	0.84		
	Book club	1.72	0.97		
DS	Physical activity	−1.25	0.88	1.568	0.201
	Cognitive training	−1.52	1.15		
	Combined training	−1.01	0.73		
	Book club	−1.27	0.84		
GC	Physical activity	−0.22	1.00	1.345	0.263
	Cognitive training	−0.42	0.92		
	Combined training	−0.01	0.96		
	Book club	−0.46	1.02		
GVM	Physical activity	−0.33	1.21	1.138	0.337
	Cognitive training	−0.35	1.03		
	Combined training	0.10	1.14		
	Book club	−0.09	1.08		
SVP	Physical activity	−0.40	1.12	0.188	0.905
	Cognitive training	−0.36	1.02		
	Combined training	−0.23	1.02		
	Book club	−0.27	0.73		
IN	Physical activity	0.26	1.75	0.527	0.664
	Cognitive training	0.26	1.08		
	Combined training	−0.04	0.76		
	Book club	0.00	1.07		
NM	Physical activity	−0.02	0.83	0.043	0.988
	Cognitive training	−0.10	0.85		
	Combined training	−0.08	1.10		
	Book club	−0.05	0.69		
PL	Physical activity	0.32	1.12	0.018	0.997
	Cognitive training	0.31	1.22		
	Combined training	0.28	1.06		
	Book club	0.35	1.21		
SH	Physical activity	−0.01	0.76	0.849	0.470
	Cognitive training	0.22	0.73		
	Combined training	−0.05	0.89		
	Book club	−0.04	0.80		
TE	Physical activity	−0.30	1.19	0.615	0.607
	Cognitive training	−0.07	0.78		
	Combined training	−0.13	1.08		
	Book club	−0.38	1.03		
VS	Physical activity	0.00	1.35	1.192	0.316
	Cognitive training	−0.32	1.24		
	Combined training	0.20	1.05		
	Book club	−0.22	0.99		
WM	Physical activity	−0.37	1.19	1.242	0.298
	Cognitive training	−0.33	1.01		
	Combined training	0.09	1.11		
	Book club	−0.02	1.10		

*AM, auditory (non-linguistic) working memory; AW, self-awareness; DA, divided attention; DS, avoiding distracters; GC, hand-eye coordination; GVM, global visual memory; IN, inhibition; NM, naming; PL, planning; SH, shifting; SVP, speed of visual-spatial information processing; TE, time estimation; VS, visual scanning; WM, verbal auditory working memory*.

**Table 8:** **Means and standard deviations at baseline and post-training for the four study groups**.

	**Cognitive training**						**No cognitive training**					
	**No physical training (*n* = 33)**		**Physical training (*n* = 29)**		**Total (*n* = 62)**		**No physical training (*n* = 29)**		**Physical training (*n* = 31)**		**Total (*n* = 60)**	
	***M***	***SD***	***M***	***SD***	***M***	***SD***	***M***	***SD***	***M***	***SD***	***M***	***SD***
AM1	−0.06	0.93	−0.21	0.82	−0.13	0.88	−0.26	1.17	−0.28	0.93	−0.27	1.05
AM2	0.08	0.08	0.00	0.74	0.04	0.75	−0.17	0.62	−0.08	0.64	−0.13	0.63
AW	−0.39	0.92	−0.07	1.03	−0.24	0.98	−0.42	0.91	0.04	1.02	−0.18	0.99
AW2	−0.29	0.93	−0.13	0.97	−0.21	0.94	−0.40	0.71	−0.14	0.90	−0.27	0.82
DA	1.57	1.26	1.92	0.84	1.73	1.09	1.72	0.97	1.76	1.03	1.74	1.00
DA2	2.32	0.89	2.14	0.82	2.23	0.85	1.79	1.00	1.91	1.43	1.85	1.24
DS	−1.52	1.15	−1.01	0.73	−1.28	1.00	−1.27	0.84	−1.25	0.88	−1.26	0.85
DS2	−0.82	0.64	−0.82	0.65	−0.82	0.64	−1.22	0.96	−1.04	1.03	−1.12	0.99
GC	−0.42	0.92	−0.01	0.96	−0.23	0.96	−0.46	1.02	−0.22	1.00	−0.34	1.01
GC2	0.30	0.87	0.77	0.97	0.52	0.94	−0.45	1.03	−0.08	1.02	−0.26	1.03
GVM1	−0.35	1.03	0.10	1.14	−0.14	1.10	−0.09	1.08	−0.33	1.21	−0.22	1.15
GVM2	0.27	0.90	0.48	1.33	0.37	1.12	−0.12	0.97	−0.23	0.98	−0.17	0.97
IN	0.26	1.08	−0.04	0.76	0.12	0.95	0.00	1.07	0.26	1.75	0.13	1.45
IN2	0.09	1.18	0.10	0.80	0.10	1.01	0.09	0.83	−0.10	0.48	0.00	0.67
NM	−0.10	0.85	−0.08	1.10	−0.09	0.97	−0.05	0.69	−0.02	0.83	−0.04	0.76
NM2	0.51	0.62	0.46	0.86	0.48	0.73	0.01	0.88	0.18	0.87	0.10	0.87
PL	0.31	1.22	0.28	1.06	0.29	1.14	0.35	1.21	0.32	1.12	0.33	1.15
PL2	0.18	0.92	−0.10	0.80	0.05	0.87	0.34	1.30	0.36	1.09	0.35	1.19
SH	0.22	0.73	−0.05	0.89	0.10	0.82	−0.04	0.80	−0.01	0.76	−0.02	0.77
SH2	0.35	0.69	0.33	0.80	0.34	0.74	0.05	0.80	0.14	0.79	0.10	0.79
SVP1	−0.36	1.02	−0.23	1.02	−0.30	1.02	−0.27	0.73	−0.40	1.12	−0.34	0.95
SVP2	0.23	0.57	0.30	0.60	0.26	0.58	−0.25	0.69	−0.34	0.94	−0.29	0.82
TE	−0.07	0.78	−0.13	1.08	−0.10	0.92	−0.38	1.03	−0.30	1.19	−0.34	1.11
TE2	0.17	1.04	0.13	0.96	0.15	0.99	−0.25	0.84	−0.13	0.83	−0.19	0.83
VS	−0.32	1.24	0.20	1.05	−0.08	1.18	−0.22	0.99	0.00	1.35	−0.11	1.19
VS2	0.44	0.66	0.47	0.68	0.45	0.66	−0.14	0.95	0.00	1.00	−0.06	0.97

*AM, auditory (non-linguistic) working memory; AW, self-awareness; DA, divided attention; DS, avoiding distracters; GC, hand-eye coordination; GVM, global visual memory; IN, inhibition; NM, naming; PL, planning; SH, shifting; SVP, speed of visual-spatial information processing; TE, time estimation; VS, visual scanning*.

**Table 9:** ***F* statistics for the interactions in the repeated measures analyses**.

	**Time × cognitive training**			**Time × physical training**		**Time × cognitive training × physical training**	
	***F*_(1, 117)_**	***p***	**Cohen's-*d***	***F*_(1, 117)_**	***p***	***F*_(1, 117)_**	***p***
AM	0.05	0.825		0.39	0.531	0.01	0.946
AW	0.07	0.798		1.07	0.302	0.02	0.894
DA	2.10	0.150		0.87	0.354	1.57	0.212
DS	1.85	0.177		0.81	0.370	3.24	0.074
GC	57.60	0.000	0.80	1.07	0.302	0.73	0.396
IN	0.18	0.670		0.10	0.751	2.14	0.146
NM	8.12	0.005	0.82	0.04	0.849	0.70	0.404
PL	1.15	0.286		0.16	0.694	0.32	0.574
SVP	23.52	0.000	0.71	0.02	0.878	0.43	0.511
SH	0.79	0.376		1.29	0.258	0.41	0.526
TE	0.14	0.706		0.01	0.944	0.09	0.767
VS	5.46	0.021	0.77	2.14	0.147	1.11	0.294
GVM	9.48	0.003	0.64	0.12	0.731	1.43	0.234

*AM, auditory (non-linguistic) working memory; AW, self-awareness; DA, divided attention; DS, avoiding distracters; GC, hand-eye coordination; GVM, global visual memory; IN, inhibition; NM, naming; PL, planning; SH, shifting; SVP, speed of visual-spatial information processing; TE, time estimation; VS, visual scanning*.

When compared to participants who did not receive cognitive training (all participants in the control group and participants in the physical training group), participants who received cognitive training, (all participants in the cognitive training intervention and in the combined physical-cognitive intervention) improved on several verbal and non-verbal cognitive abilities (Table 9): Hand-Eye Coordination, GVM (working memory and long-term memory), Speed of Information Processing, Visual Scanning, and Naming. Cohen's-*d*, calculated for these improvements revealed medium-size (*d* = 0.6 or 0.7) or large-size effects (*d* = 0.8). None of the three way interactions reached statistical significance. Essentially the same results were obtained when health was added as a second covariate (Hand-Eye Coordination *F* = 61.038, *p* = 0.000; Naming *F* = 8.790, *p* = 0.004; SVP *F* = 23.083, *p* = 0.000; Visual Scanning *F* = 5.401, *p* = 0.022; and GVM *F* = 10.102, *p* = 0.002).

#### Within group differences

After adjusting the alpha level for test multiplicity by dividing the accepted alpha (0.05) by 13 (the number of *t*-tests conducted), we found that the cognitive training group had improved significantly from baseline to post-testing on Divided Attention (*t* = −3.48; *p* = 0.001), Avoiding Distractions (*t* = −3.59; *p* = 0.001), Hand-eye Co-ordination (*t* = −10.84; *p* = 0.000), Naming (*t* = −5.66; *p* = 0.000), Speed of Visual-Spatial Information Processing (*t* = −5.17; *p* = 0.000), Visual Scanning (*t* = −3.41; *p* = 0.002), and Global Visual Memory (*t* = −4.56; *p* = 0.000).

After adjusting alpha for multiple tests, we found that fewer significant improvements are observed in the combined intervention group. This group improved on Eye-Hand Coordination (*t* = −9.602; *p* = 0.000), Naming (*t* = −3.246; *p* = 0.003), and SVP (*t* = −4.695; *p* = 0.000).

Neither the physical activity group nor the book-reading club showed any improvement on any of the 13 cognitive abilities. Uncorrected alpha levels for these groups range from 0.094 to 0.983. These results, which explain the absence of significant three-way-interactions (TIME × COGNITIVE TRAINING × PHYSICAL ACTIVITY), suggest that the few improvements observed in the combined intervention group are driven solely by the cognitive training.

## Discussion

Based on previous research, we hypothesized that an intervention combining physical activity with cognitive training would yield significantly larger cognitive benefits than single-domain interventions which trained physical activity or cognitive training separately. We found that the two groups of older persons that engaged in cognitive training (separately or combined) significantly improved their memory, processing speed, eye-hand coordination, naming, and visual-spatial processing ability scores. We observed no such improvements in the groups that did not engage in cognitive training. These differences and improvements held true not only between groups but also within groups.

Cognitive training using the CogniFit program has previously yielded improved cognitive ability scores in healthy older adults (Verghese et al., 2010; Peretz et al., 2011) as well as in impaired populations (Horowitz-Kraus and Breznitz, 2009; Shatil et al., 2010; Haimov and Shatil, in press), and the present results are further evidence that personalized and systematic cognitive training taps into brain plasticity and appears to induce changes in cognitive function that are captured by improved scores on tasks measuring cognitive ability. While the effects for Speed of Visual-Spatial Information Processing, Visual Scanning, and Global Visual Memory were of medium size, those for Naming and Hand-Eye Coordination were large. In the Peretz et al. (2011) study, also conducted in a population of healthy older adults, the same cognitive training program was significantly superior to computer games in improving scores on visuospatial learning and visuospatial working memory tasks, two processes that are important for daily tasks (Maeshima et al., 1997). Similar results emerge from the present study. This replication of findings is noteworthy, especially given that visuospatial processing is known to deteriorate with age (Kemps and Newson, 2006) and is not normally considered a fluid ability.

The present study did not extend to everyday tasks and did not investigate whether the observed benefits endure beyond the period of cognitive training. However, there are indications in the literature that cognitive training improves health-related quality of life (Wolinsky et al., 2006) and activities of daily living (Willis et al., 2006), and that gains persist in the long-term (Willis et al., 2006; Wolinsky et al., 2006). Improvements in speed of processing are related in a previous study (Ball et al., 2007) to benefits in the Useful Field of View test, itself, a good predictor of driving. Also, in the same study, benefits in speed of processing due to training were maintained for at least 2 years, and translated to improvements in everyday abilities, including performance of instrumental activities of daily living and safer driving. Our cognitive training groups also improved on Eye-Hand Coordination (required for driving) and on naming words, a well-documented deficit in the elderly (James, 2006; Burke and Shafto, 2008). Taken together, the cognitive gains in the cognitive training group may potentially impact quality of life and the wide range of verbal and non-verbal daily functions associated with cognitive training in the above studies.

Surprisingly, the group that engaged in physical activity only, showed no such cognitive effects. These findings contradict a research consensus that aerobic activity is a main mechanism in the enhancement of cognitive ability (Kramer et al., 1999). Aerobic activity, which was purposefully kept mild due to the advanced age of more than half the participants, could have been insufficiently represented in our study. Also, our study lasted 4 months but there is some indication that aerobic training (principally brisk-walking) must be practiced for at least one consecutive year to produce cognitive benefits in sedentary older adults (Voss et al., 2010). Thus, both the nature and the duration of aerobic regimen may be important factors for the manifestation of cognitive gains.

As in the present study, Oswald et al. (2006) failed to find cognitive gains in the group engaged in physical activity alone but not in the group engaged in the combined intervention. They explained the advantages conferred by the combined interventions by referring to the beneficial effect of physical activity on brain metabolism, but suggest that this metabolic benefit can be put to use only if a cognitive effort must be invested (like the effort required by cognitive training, for example). In the present study, the lack of a significant three-way interaction does not support Oswald et al.'s interpretation but rather points to cognitive training as the main agent for cognitive changes. This possibility raises the hope that cognitive training might prove beneficial to older adults who are not able to engage in regular aerobic activity.

Interesting patterns of adherence emerged from this study. First, 91 of 94 non-completers abandoned the study *before* the training activities had begun, either immediately after providing informed consent or during baseline. Baseline testing lasted several days and included neuropsychological tests, demographic and quality of life questionnaires, and physical fitness tests (not reported here). This period might have been a good index by which to assess the time and health limitations imposed by the interventions. Indeed, the most frequent reasons for leaving the study at this earliest stage were poor health or heavy occupation load. Second, no non-completer gave both reasons for leaving, suggesting that in this study non-completers constitute one group occupied with self-rewarding, time-consuming activities, and another group incapacitated by health problems. The latter group makes up 48.2% of all non-completers when non-participation caused by spouses' poor health is included. These data emphasize the link between poor physical health of the participant (and his or her life partner) and reduced opportunity to participate in stimulating activity. Once the training activities began, only three people left the study, all for health-related reasons. This strengthens our suggestion that poor physical health emerges as a main incapacitating factor in this study. Non-completers also appeared to be less educated than completers but this finding is impaired by incomplete education data among non-completers and by the generally high educational level among the Lakeview residents.

Furthermore, the high adherence rate observed in all groups following baseline testing suggests that the social support afforded by the community (coordinators, members in the intervention groups, and volunteers) could have been determinant. This possibility has led to improvements in the cognitive training system used for this study (the delivery platform now allows for extensive, varied interactions among trainees, a feature not in existence prior to this study).

How would adherence and perseverance be affected without support, encouragement, instructors, and modern facilities? Unprompted adherence to a home-based physical activity regimen remains undocumented. Unprompted adherence using the same cognitive training as used here indicate that adherence patterns (3.2% of sample quit after beginning training) were quite similar in older healthy individuals self-training at home (Peretz et al., 2011). Adherence was much lower among individuals with multiple sclerosis also self-training at home, where 34% of the participants left the study (Shatil et al., 2010). Peer-group support and instructor mediation might prove beneficial for the more health-vulnerable individuals.

Based on the present results, some recommendations could be made for researchers planning prevention trials, for example, on Alzheimer's disease. It would be important to adopt an aerobic regimen which is individually matched to the patient's physical health and fitness, so that highly fit individuals might train longer, using more challenging aerobic tasks while less fit participants might train with milder aerobic activity. A range of aerobic exercises should be developed that would be performed while sitting, in order to accommodate the oldest participants and those most at risk for falls. Another recommendation for such trials would be the need to increase aerobic challenge for all subjects as the study progresses. In our trial both requirements (personalization of training and rising difficulty levels) were fulfilled with regard to the cognitive training program but not with regard to the physical activity training. In retrospect, this was a limitation of the study. Researchers should carefully plan the length of training and aim for a period of one year in the case of aerobic training. For better results a social interaction component should be embedded in the training delivery. Policy makers should be made fully aware of the potential that such interventions hold for the elderly and should encourage their continued investigation, in isolation or combined with other life-endowing components such as nutrition and mood.

Despite the main results in this investigation, one must emphasize the extreme necessity of physical activity for the elderly. Research on older adults is unanimous in linking physical activity to several physiological (Anderson et al., 2010) and neurological (Motl et al., 2008) gains; to increases in longevity (Erikssen et al., 1998; Hu et al., 2004) and in conferring important protective (Colcombe et al., 2003) and regenerative (Colcombe et al., 2006) defense against cognitive decline, so that a sedentary life and one of physical immobility would prove detrimental to a person's cognitive and physical health, regardless of whether that person engaged in cognitive training.

The main strengths of the present study are a 4 group-design, a well-distributed social support across all intervention groups, an extended duration of the intervention (4 full months, individual sessions of 40 min each). The limitations of the study include a possibly insufficient duration of aerobic training, the lack of health, functional, and quality-of-life endpoints to evaluate any associations with benefits in daily living or quality of life impact, and no post-intervention follow-up to assess the maintenance of observed improvements.

Future studies should continue to investigate the effects on cognitive function of both combined and isolated cognitive training and physical activity training. Research should be carried out to inform concretely on what constitutes an adequate aerobic regimen for improving cognitive function in older adults.

### Conflict of interest statement

Dr. Evelyn Shatil is an employee at CogniFit Inc.
